# Multiday acute sodium bicarbonate intake improves endurance capacity and reduces acidosis in men

**DOI:** 10.1186/1550-2783-10-16

**Published:** 2013-03-26

**Authors:** Sandro Manuel Mueller, Saskia Maria Gehrig, Sebastian Frese, Carsten Alexander Wagner, Urs Boutellier, Marco Toigo

**Affiliations:** 1 Exercise Physiology, Institute of Human Movement Sciences, ETH Zurich, Zurich, Switzerland; 2 Department of Neurology, University Hospital Zurich, Zurich, Switzerland; 3 Institute of Physiology, University of Zurich, Zurich, Switzerland; 4 Zurich Center for Integrative Human Physiology, University of Zurich, Zurich, Switzerland

**Keywords:** Critical power, Supplementation, Plasma volume, Placebo, Endurance, Sodium

## Abstract

**Background:**

The purpose was to investigate the effects of one dose of NaHCO_3_ per day for five consecutive days on cycling time-to-exhaustion (*T*_lim_) at ‘Critical Power’ (CP) and acid–base parameters in endurance athletes.

**Methods:**

Eight trained male cyclists and triathletes completed two exercise periods in a randomized, placebo-controlled, double-blind interventional crossover investigation. Before each period, CP was determined. Afterwards, participants completed five constant-load cycling trials at CP until volitional exhaustion on five consecutive days, either after a dose of NaHCO_3_ (0.3 g·kg^-1^ body mass) or placebo (0.045 g·kg^-1^ body mass NaCl).

**Results:**

Average *T*_lim_ increased by 23.5% with NaHCO_3_ supplementation as compared to placebo (826.5 ± 180.1 *vs.* 669.0 ± 167.2 s; *P* = 0.001). However, there was no time effect for *T*_lim_ (*P* = 0.375). [HCO_3_^-^] showed a main effect for condition (NaHCO_3_: 32.5 ± 2.2 mmol·l^-1^; placebo: 26.2 ± 1.4 mmol·l^-1^; *P* < 0.001) but not for time (*P* = 0.835). NaHCO_3_ supplementation resulted in an expansion of plasma volume relative to placebo (*P* = 0.003).

**Conclusions:**

The increase in *T*_lim_ was accompanied by an increase in [HCO_3_^-^], suggesting that acidosis might be a limiting factor for exercise at CP. Prolonged NaHCO_3_ supplementation did not lead to a further increase in [HCO_3_^-^] due to the concurrent elevation in plasma volume. This may explain why *T*_lim_ remained unaltered despite the prolonged NaHCO_3_ supplementation period. Ingestion of one single NaHCO_3_ dose per day before the competition during multiday competitions or tournaments might be a valuable strategy for performance enhancement.

**Trial registration:**

Trial registration: ClinicalTrials.gov Identifier NCT01621074

## Background

Competitive sports performance is strongly dependent on optimal muscle function. During cycling exercise across the heavy and severe intensity domains [1], energy is provided more and more by anaerobic glycolysis. This leads to an increased rate of accumulation of metabolites, which have been linked with muscle fatigue (*e.g.* P_i_, ADP, H^+^, and extracellular K^+^). Cycling exercise at the threshold between the heavy and severe domain, *i.e.* at ‘Critical Power’ (CP), can, in contrast to the theoretical concept [2], only be sustained for as long as 20 to 40 min [3] before task failure. Furthermore, it was shown that CP overestimates the highest possible metabolic steady state [4,5] and, consequently, that exercise at or above CP is associated with a decline in muscle and blood pH [6,7]. An activity-induced decrease in intracellular pH has been suggested to limit exercise because it inhibits glycogenolysis and glycolysis [8], increases muscular K^+^-release [9] and inhibits sarcoplasmatic Ca^2+^-release [10,11]. Furthermore, it induces a metabolic acidosis that might impair muscle function [12] and compromise performance. To blunt the fall in intracellular pH and prolong time-to-exhaustion (*T*_lim_), nutritional modulation might be a promising avenue. With respect to endurance exercise, to date especially sodium bicarbonate (NaHCO_3_) has gained much attention. However, the mechanisms by which NaHCO_3_ ingestion may enhance performance are not fully understood. It is believed that NaHCO_3_ ingestion leads to an increase in blood bicarbonate concentration ([HCO_3_^-^]), which in turn increases extracellular buffer capacity. More precisely, it is proposed that the higher [HCO_3_^-^] gradient between blood and the intramyocellular compartment enhances H^+^-efflux out of the muscle cell, thereby delaying the fall in intracellular pH [13], which in turn may delay an impairment in optimal muscle function and performance [14,15]. Therefore, NaHCO_3_ supplementation would be expected to improve *T*_lim_ at CP if muscle pH is a limiting factor for exercise tolerance.

Basically, three types of NaHCO_3_ supplementation protocols can be applied: acute (single dose), chronic (multiple dose) and multiday acute supplementation (one dose per day before competition for consecutive days of competition). During the acute delivery mode participants take one single dose (mostly 0.3 g∙ kg^-1^ body mass NaHCO_3_) 60 to 90 min before the start of competition. During the chronic delivery mode participants take a daily amount of NaHCO_3_ (mostly 0.5 g∙ kg^-1^ body mass), divided in 2 to 3 portions, for several days before competition takes place. On the day of competition, no NaHCO_3_ is consumed [16,17]. The multiday acute delivery mode comprises the ingestion of acute doses on consecutive days of competition. In contrast to the chronic loading protocol, acid–base balance is perturbed on every day during the multiday acute delivery mode. This fact leads to major differences regarding the acid–base status and accordingly the underlying mechanisms as well as the effectiveness of the different delivery modes. While the acute and chronic supplementation protocols are scientifically well described, data on the effects of multiday acute supplementation are lacking. There are several studies, which investigated NaHCO_3_ ingestion during tournament-like sports, but only for single events. For example, it was shown that NaHCO_3_ supplementation increases tennis performance [18] but does not affect prolonged intermittent cycling exercise performance [19]. However, up to date, no study investigated the effect of a consecutive multiday supplementation on consecutive multiday performance. Since consecutive, acute-load daily use of NaHCO_3_ might represent an interesting option to increase performance during multiday competitions or tournaments that involve exercise in the heavy and severe intensity domains, further research is warranted. In particular, scientific knowledge is limited with respect to the recovery of the body’s acid–base balance after high-intensity exercise with NaHCO_3_ supplementation and consequently, the initial positions on the following days remain elusive.

Thus, the purpose of this randomized, placebo-controlled, double-blind interventional crossover study was to investigate if multiday acute NaHCO_3_ supplementation in well-trained endurance athletes leads to changes in *T*_lim_ at CP during constant-load cycle ergometer trials on a day-to-day basis with daily acute NaHCO_3_*vs.* placebo supplementation for 5 days. Furthermore, we aimed to investigate if differences in *T*_lim_ can be explained by alterations in [HCO_3_^-^] and if the high amount of ingested Na^+^ influences plasma volume (PV) and thus [HCO_3_^-^]. Given that exercise at or above CP leads to muscle and blood acidification [6,7], and that [HCO_3_^-^] increases extracellular buffer capacity [13], we hypothesized that consecutive, acute-load daily supplementation of NaHCO_3_ increases *T*_lim_ relative to placebo. We assumed that an increase in [HCO_3_^-^] after the first intake is responsible for the rise in *T*_lim_. Since during multiday NaHCO_3_ intake, a high amount of Na^+^ is ingested and absorbed, detrimental effects on endurance performance are possible. In fact, a higher [Na^+^] leads to water retention and thereby results in PV expansion [20]. An increase in PV decreases blood ion concentrations, and as such results in a diminished [HCO_3_^-^], which in turn could counteract the benefits associated with NaHCO_3_ intake. It is therefore questionable, whether [HCO_3_^-^] can be increased beyond the concentration reached after the first day of supplementation on all subsequent days of supplementation. Consequently, we hypothesized that PV expands following a high Na^+^ intake, limiting any further increase in [HCO_3_^-^], and consequently *T*_lim_, beyond that observed after the first day of supplementation.

## Methods

### Participants

Eleven well-trained male cyclists and triathletes volunteered to participate in this study. The participants were recruited from different cycling or triathlon clubs. Two of them were excluded from the analysis because they contravened our instructions. One participant did not refrain from high-intensity exercise and the other markedly increased the training volume during or before the second testing sessions (see below). Another participant had to abort the measurements because of illness. The physical characteristics of the remaining eight participants were (mean ± SD) age 31.4 ± 8.8 years, height 184.6 ± 6.5 cm, body mass 74.1 ± 7.4 kg, peak power output (*P*_peak_) during ramp test 402.0 ± 29.1 W, peak oxygen uptake (*V̇ *O_2peak_) 61.0 ± 4.3 ml∙ kg^-1^∙ min^-1^. These athletes were all involved in their early preparation phase of training (pre-season). During this phase, the training consisted of constant-load rides at low-intensity. The participants were instructed to maintain their individual, low-intensity training programs. Additionally, they were advised to refrain from any high-intensity exercise during the testing sessions and to continue their nutritional habits. The determination of CP after the wash-out phase served to ascertain that no training effect occurred during the first phase of the study. None of the participants included was currently using buffer substances or any other ergogenic agents that may have compromised the administration of NaHCO_3_. Participants were fully informed about the purposes, benefits and risks associated with this study and completed a routine health questionnaire before giving written informed consent. This study was approved by the Swiss Federal Institute of Technology Zurich (ETH) ethics committee and was conducted in accordance with the Declaration of Helsinki.

### Experimental overview

**Figure 1:**
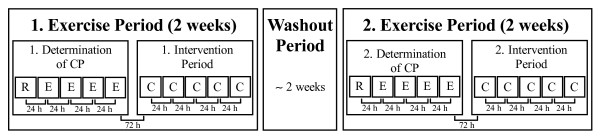
**Study design.** C, constant-load trials at ‘Critical Power’ (CP); E, constant load tests; R, incremental ramp test.

Using a randomized, placebo-controlled, double-blind interventional crossover design, all participants completed two exercise periods, each consisting of ten testing sessions (Figure 1). These periods were separated by at least one week and on average 2.3 ± 2.1 weeks of washout, during which the participants maintained their low-intensity training programs. During both periods, the first five tests were conducted to determine CP and consisted of one incremental test and four constant-load tests to volitional exhaustion. The determination of CP was followed by a five-day intervention period, which was conducted either with NaHCO_3_ or sodium chloride (NaCl) supplementation. On each day during the intervention period, a constant-load trial at CP was performed. All tests were carried out under temperature-controlled laboratory conditions (19–24°C) and at the same time of day. The participants had a 23 h 34 min ± 53 min and 23 h 22 min ± 45 min rest period between the single tests during the placebo and NaHCO_3_ trials, respectively. All test devices were calibrated before, and whenever indicated after each test under the terms of the manufacturer’s recommendations. An independent researcher randomly assigned the two conditions to the participants and administered the non-distinguishable placebo or NaHCO_3_ tablets without revealing the ingredient. The investigator performing the tests was also blinded to the treatment. No feedback on test performance was given to the participants until all trials had been finished.

### Supplementation

NaHCO_3_ was administered orally as tablets (Bullrich Salz Magentabletten, delta pronatura Dr. Krauss & Dr. Beckmann, Egelsbach, Germany). The NaHCO_3_ and placebo tablets (NaCl, delta pronatura Dr. Krauss & Dr. Beckmann, Egelsbach, Germany) were matched by shape and taste. During the two conditions either 0.3 g·kg^-1^ body mass of NaHCO_3_ or 0.045 g∙ kg ^-1^ body mass of NaCl [21,22] had to be ingested 90 min before [17] each of the five consecutive constant-load trials. Each supplement was consumed during a 15-min period with 0.75 dm^3^ still water to minimize gastrointestinal discomfort or any other adverse effects [8,23]. One NaHCO_3_ tablet contained 850 mg of NaHCO_3_, whereas one placebo tablet contained 130 mg of NaCl, which assured the intake of equal number of pills during the varying conditions (*i.e.* 0.35 tablets∙ kg^-1^ body mass). If a participant’s body mass was such that they required to consume a non-round number of tablets, the participants were instructed to consume the number of pills rounded to the nearest whole pill required to obtain the dose. To minimize falsification of the pill count, participants were given an unknown (to them) number of pills in excess of needs and were asked to return any remaining pills at the end of the study.

### Determination of ‘critical power’

Five cycle ergometer tests were performed to determine CP [24]. On the first visit, the seat and handle bar of the cycle ergometer (Ergoselect 200 K, Ergoline, Bitz, Germany) were adjusted. These settings were adopted for all consecutive trials. Participants started with a ramp cycle ergometer test to determine *P*_peak_ and *V̇ *O_2peak_. After a 3-min rest, the ramp test started at 100 W and involved power increases of 9 W every 18 s (30 W∙ min^-1^) until volitional exhaustion. For all tests, participants were asked to maintain a cadence of 80 revolutions per min throughout the test. Volitional exhaustion, *i.e.* task failure, for all cycling tests was defined as the point in time when participants stopped pedaling or the cadence fell below 75 revolutions per minute for > 5 s. On each of the following testing days, one constant-load trial at different power output was completed to determine CP. After a 3-min rest, participants started with a 5-min warm-up at 75 W [25]. The power was then increased immediately to 85%, 90%, 95% or 105% of *P*_peak_ in a randomized order (modified from Brickley *et al.*[25] including the 85% stage). These endurance capacity tests were conducted until task failure. Using the *T*_lim_ from these tests, CP was then calculated from the linear power-time^-1^ equation [24].

### Constant-load cycling trials at ‘critical power’

During each of the two intervention periods, five constant-load trials at CP were completed on five consecutive days. These trials started with a 3-min rest and were followed by a 5-min warm-up at 75 W. Subsequently, power was immediately increased to the previously calculated CP and participants were encouraged to maintain the given cadence for as long as possible.

### Gas exchange and heart rate analysis

Participants were equipped with a facemask, which covered their mouth and nose (Hans Rudolph, Shawnee, KS, USA). The facemask was connected with an anti-bacterial filter (PALL PRO1087, Pall, East Hills, NY, USA) to an Innocor™ device (Innocor™, Innovision, Odense, Denmark). Pulmonary gas exchange and ventilation were continuously measured breath by breath throughout all ergometer trials. Throughout all cycling tests, heart rate was recorded (Polar S610i, Polar Electro, Kempele, Finland). *V̇ *O_2peak_, *V̇ *O_2_ during the constant-load trials at CP (*V̇ *O_2,CLT_), carbon dioxide output during the constant-load trials at CP (*V̇ *CO_2,CLT_), respiratory exchange ratio during the constant-load trials at CP (RER_CLT_) and heart rate during the constant-load trials at CP (HR_CLT_) were determined as the highest mean over a 10-s period. The *V̇ *O_2_ slow component was calculated as the difference between the changes in *V̇ *O_2_ between min 2 and task failure and between min 2 and 6.

### Blood analysis

For the analysis of [HCO_3_^-^], [Na^+^], pH and actual base excess (ABE) 125 μl blood from the same earlobe were always obtained 75 min after the NaHCO_3_ ingestions and 15 min before the constant-load trials at CP on 1 and day 5. Blood was collected in a heparinized glass capillary tube and analyzed using a clinical blood gas analyzer (ABL 505, Radiometer, Copenhagen, Denmark). Venous blood samples (4 ml) were collected from the cubital vein before the constant-load trials at CP on days 1 and 5 (medica, Medizinische Laboratorien Dr. F. Kaeppeli, Zurich, Switzerland). These blood samples were analyzed for hemoglobin concentration and hematocrit, which were used to calculate changes in PV according to Dill and Costill [26].

### Body composition measurement

A densitometer (Lunar iDXA™, GE Healthcare, Madison, WI, USA) was used for the determination of total lean body mass and lean soft tissue mass of the legs. Dual-energy X-ray absorptiometry (DXA) measurements were performed just before the constant-load trials every second day throughout the intervention periods to assess leg lean mass as an indicator of glycogen content. According to the DXA two-component soft tissue model, lean soft tissue mainly consists of water, proteins, glycogen and soft tissue minerals [27]. Water and glycogen content are further interconnected since each gram of glycogen binds 3–4 g of water [28]. To ensure a similar provision of carbohydrates in the immediate post-exercise period, participants were given 0.75 dm^3^ of a regeneration drink (57 g carbohydrates∙ portion^-1^, Carbo Basic Plus, Winforce, Menzingen, Switzerland) instantly after completion of each constant-load trial.

### Statistical analysis

To assess differences in *T*_lim_, blood values, gas exchange, heart rate, and body composition a two-way repeated-measures ANOVA having two levels of condition (NaHCO_3_ and placebo) and five levels of time (5 days of testing) was used. The assumption of sphericity was tested using Mauchly’s test. If the assumption of sphericity was violated, the degrees of freedom were corrected using the Greenhouse-Geisser estimates of sphericity. When *F* ratios were significant, *post hoc* comparisons of main effects were performed using a Student’s paired *t*-test with Bonferroni correction. PV data were not normally distributed and thus log-transformed before using the described analysis. All data are presented as means ± SD. The effect size is denoted as η_p_^2^ (partial eta-squared). The level of significance was set at *P* < 0.05. The statistical analyses were conducted using the software SPSS Statistics 20.0 (SPSS, Chicago, IL, USA).

## Results

**Figure 2:**
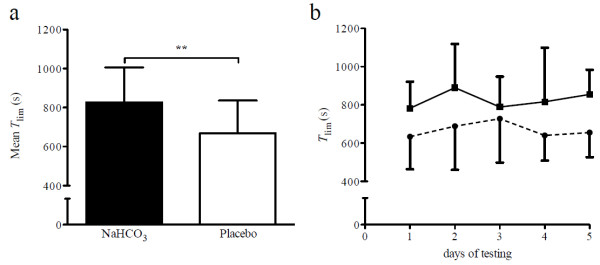
**Time-to-exhaustion with NaHCO₃ and placebo supplementation. a)** Mean ± SD time-to-exhaustion (*T*_*lim*_) with NaHCO_3_ and placebo, respectively, **P < 0.01; **b)***T*_*lim*_ with NaHCO_3_ (solid line) and placebo (dashed line) on the 5 days of testing are presented as group mean ± SD (*n* = 8).

As judged by the leftover pill count, average compliance with NaHCO_3_ and placebo supplementation was 100%. *T*_lim_ increased by 23.5% following NaHCO_3_ ingestion (*F*_(1,7)_ = 35.45, *P* = 0.001, η_p_^2^ = 0.84; Figure 2a). However, there was neither an effect of time (*F*_(4,28)_ = 1.1, *P* = 0.375, η_p_^2^ = 0.14) nor an intervention x time interaction (*F*_(4,28)_ = 0.74, *P* = 0.464, η_p_^2^ = 0.01; Figure 2b). No differences in CP, as measured before the first and second supplementation period, could be found (306.8 ± 21.4 W *vs.* 309.0 ± 30.4 W; *F*_(1,7)_ = 0.15, *P* = 0.708, η_p_^2^ = 0.02). Also, no difference could be found between CP as determined before the NaHCO_3_ and placebo intervention (304.3 ± 25.6 W *vs.* 311.5 ± 26.5 W; *F*_(1,7)_ = 1.99, *P* = 0.202, η_p_^2^ = 0.22).

The NaHCO_3_ intervention resulted in a significantly higher [HCO_3_^-^] relative to placebo (*F*_(1,7)_ = 118.71, *P* < 0.001, η_p_^2^ = 0.94; Table 1). However, there was neither a main effect for time (*F*_(1,7)_ = 0.05, *P* = 0.835, η_p_^2^ = 0.01) nor an intervention x time interaction (*F*_(1,7)_ = 0.04, *P* = 0.855, η_p_^2^ = 0.01). [Na^+^] increased after NaHCO_3_ (*F*_(1,7)_ = 12.44, *P* = 0.012, η_p_^2^ = 0.68) but remained constant with placebo supplementation. [Na^+^] did not significantly change over time (*F*_(1,7)_ = 0.49, *P* = 0.509, η_p_^2^ = 0.08) with either condition. The mean ABE were significantly higher during the NaHCO_3_ compared to the placebo trials (*F*_(1,7)_ = 100.42, *P* < 0.001, η_p_^2^ = 0.94), but not between days of testing (*F*_(1,7)_ = 0.01, *P* = 0.920, η_p_^2^ = 0.00). Blood pH was increased with NaHCO_3_ supplementation (*F*_(1,7)_ = 42.04, *P* < 0.001, η_p_^2^ = 0.86), showing no change between the testing days (*F*_(1,7)_ = 1.11, *P* = 0.327, η_p_^2^ = 0.14). There was a main effect for a PV increase during interventions (*F*_(1,7)_ = 19.22, *P* = 0.003, η_p_^2^ = 0.73; Table 1) and days of testing (*F*_(1,7)_ = 18.12, *P* = 0.004, η_p_^2^ = 0.72), as well as a significant intervention x time interaction (*F*_(1,7)_ = 22.05, *P* = 0.002, η_p_^2^ = 0.76).

**Table 1:** **[HCO**_**3**_^**-**^**], [Na**^**+**^**], ABE, pH and PV 75 min after supplement ingestion on the first and the fifth day of testing with either NaHCO**_**3 **_**or placebo supplementation**

	**NaHCO**_**3**_		**Placebo**	
	**Day 1**	**Day 5**	**Day 1**	**Day 5**
[HCO_3_^-^] (mmol &z.ccirf;l^-1^)	32.4 ± 1.8***	32.6 ± 2.7***	26.4 ± 1.8	26.0 ± 1.1
[Na^+^] (mmol &z.ccirf;l^-1^)	142.1 ± 3.9*	142.4 ± 3.0*	138.1 ± 1.2	139.3 ± 5.5
ABE (mmol &z.ccirf;l^-1^)	8.4 ± 1.7***	8.3 ± 2.3***	2.7 ± 1.7	2.0 ± 0.9
pH	7.49 ± 0.02***	7.48 ± 0.02***	7.44 ± 0.02	7.43 ± 0.02
PV (%)	55.5 ± 2.3	62.6 ± 3.8^††^	56.0 ± 1.7	55.9 ± 3.3

Values are mean ± SD (*n* = 8). [HCO_3_^-^], blood bicarbonate concentration; [Na^+^], blood sodium concentration; ABE, actual base excess; PV, plasma volume.

*P < 0.05, *** P < 0.001 relative to placebo at the same time point; ^††^*P* < 0.01 relative to day 1.

The NaHCO_3_ ingestion resulted in a significant intervention x time interaction for total lean body mass (*F*_(1,7)_ = 7.77, *P* = 0.027, η_p_^2^ = 0.53; Table 2). In addition, total lean body mass raised over the five consecutive testing days in both conditions (*F*_(2,14)_ = 10.97, *P* = 0.001, η_p_^2^ = 0.61; Table 2). Lean soft tissue mass of the legs did not change neither during the interventions (*F*_(1,7)_ = 3.16, *P* = 0.119, η_p_^2^ = 0.31) nor across the days of testing (*F*_(2,14)_ = 1.38, *P* = 0.283, η_p_^2^ = 0.17; Table 2).

**Table 2:** **Total lean body mass and lean soft tissue mass of the legs on the different days of testing with either NaHCO**_**3 **_**or placebo ingestion**

	**NaHCO**_**3**_			**Placebo**		
	**Day 1**	**Day 3**	**Day 5**	**Day 1**	**Day 3**	**Day 5**
Total lean soft tissue (kg)	60.7 ± 4.8	61.7 ± 5.3*^††^	62.0 ± 5.3*^††^	60.5 ± 5.3	61.3 ± 5.4^††^	60.6 ± 5.0
Lean soft tissue legs (kg)	21.1 ± 2.5	21.3 ± 2.9	21.4 ± 3.0	21.0 ± 2.9	21.2 ± 2.9	21.0 ± 2.9

Values are mean ± SD (*n* = 8). *P < 0.05 relative to placebo; ^††^*P* < 0.01 relative to day 1.

*V̇ *O_2,CLT_ and *V̇ *CO_2,CLT_ did not differ between the interventions (*F*_(1,7)_ = 1.453, *P* = 0.267, η_p_^2^ = 0.17 and *F*_(1,7)_ = 1.132, *P* = 0.323, η_p_^2^ = 0.14; Table 3) or between the days of testing (*F*_(2,14)_ = 0.631, *P* = 0.667, η_p_^2^ = 0.39 and *F*_(2,14)_ = 0.145, *P* = 0.964, η_p_^2^ = 0.020). None of the daily *V̇ *O_2,CLT_ (data not shown) differed from *V̇ *O_2peak_ (*F*_(2,14)_ = 0.081, *P* = 0.923, η_p_^2^ = 0.011). There was no difference in the *V̇ *O_2_ slow component between the NaHCO_3_ and placebo intervention (0.08 ± 0.31 vs. 0.03 ± 0.28 l∙ min^-1^ for the NaHCO_3_ and placebo intervention, respectively; *P* = 0.504). RER_CLT_ also was not different between interventions (*F*_(1,7)_ = 2.947, *P* = 0.130, η_p_^2^ = 0.30) and days of testing (*F*_(2,14)_ = 0.821, *P* = 0.523, η_p_^2^ = 0.11). HR_CLT_ decreased during the 5 testing days (*F*_(4,28)_ = 5.97, *P* = 0.001, η_p_^2^ = 0.46; Table 3) but there was no main effect for condition (*F*_(1,7)_ = 0.04, *P* = 0.852, η_p_^2^ = 0.01).

**Table 3:** **Peak values during the CLT at CP for *V*O**_**2**_**, *V***CO_2_, RER and HR on the first and fifth day of testing with either NaHCO_**3 **_**or placebo supplementation**

	**NaHCO**_**3**_		**Placebo**	
	**Day 1**	**Day 5**	**Day 1**	**Day 5**
*V*O_2,CLT_	4.64 ± 0.39	4.66 ± 0.30	4.59 ± 0.37	4.64 ± 0.47
*V*CO_2,CLT_	4.63 ± 0.47	4.67 ± 0.19	4.58 ± 0.36	4.59 ± 0.40
RER_CLT_	1.07 ± 0.04	1.08 ± 0.05	1.03 ± 0.05	1.05 ± 0.05
HR_CLT_	177.4 ± 8.5	172.8 ± 9.0**	176.3 ± 7.8	173.8 ± 8.6**

Values are mean ± SD (*n* = 8). CLT, constant-load trials; CP, ‘Critical Power’; *V*O_2_, oxygen uptake; *V*CO_2_ carbon dioxide output; RER, respiratory exchange ratio; HR, heart rate. ** *P* < 0.01 relative to day 1.

## Discussion

Several new findings have been observed in this randomized, placebo-controlled, double-blind interventional crossover investigation. First, multiday NaHCO_3_ supplementation for 5 days increased *T*_lim_ at CP on each day relative to placebo in highly trained athletes. Second, there was no difference in the increased *T*_lim_ over the 5 days of supplementation with NaHCO_3_ or NaCl. Third, the increase in *T*_lim_ was paralleled by increases in [HCO_3_^-^], pH and ABE. Fourth, [HCO_3_^-^] and [Na^+^] in the blood stabilized over time in the NaHCO_3_ condition. Fifth, calculated PV increased during the NaHCO_3_ more than in the placebo intervention.

We found that NaHCO_3_ supplementation led to an increase in *T*_lim_ at CP and that the improvement in *T*_lim_ was paralleled by an increase in blood [HCO_3_^-^], pH and ABE, indicating that the alteration in *T*_lim_ appears to be linked to an elevated extracellular buffer capacity. In fact, it has been shown that an increased [HCO_3_^-^] gradient between the intra- and extramyocellular compartment leads to an amplified H^+^-efflux from the muscle cell and delays the fall in intramyocellular pH [8,14]. We observed a trend for higher [La^-^] during the constant-load tests following NaHCO_3_ supplementation (*P* = 0.070, data not shown), supporting the notion that the increased H^+^-concentration resulted from a lactate-proton symport. A fall in intramyocellular [H^+^] is associated with muscle fatigue due to 1) an inhibition of glycogenolysis and glycolysis [8], 2) increased muscular K^+^ release, 3) lesser contractility of the heart muscle [9], 4) inhibition of the sarcoplasmatic calcium release [10] and 5) inhibition of the actin-myosin interactions [11]. Thus, delaying the fall in intramyocellular pH might postpone the fatigue process and prolong intact muscle function. Indeed, our results showed that the ingestion of NaHCO_3_ induced metabolic alkalosis, which in turn enhanced *T*_lim_ at CP and thus improved high-intensity exercise in the range of 10 to 20 min duration.

As hypothesized, *T*_lim_ at CP could be increased with NaHCO_3_ supplementation. This is in contrast to the theoretical model, which states that an intramyocellular metabolic steady state exists at exercise intensities up to CP. However, our results support the notion that CP overestimates the metabolic steady state [4,5]. Furthermore, our result that NaHCO_3_ increased *T*_lim_ at CP extends previous findings showing that NaHCO_3_ supplementation increases exercise above CP relative to placebo [14,29]. In the latter studies, short high-intensity tests, during which intramyocellular pH falls rapidly from the beginning of exercise, were completed. During these types of tests, the finite work capacity above CP (*W*^*′*^) is drawn on after the start of exercise and becomes reduced. In light of our findings, these results might be interpreted to mean that NaHCO_3_ simply increases *W’*. However, Vanhatalo *et al.*[23] showed that NaHCO_3_ does not increase *W’* during a 3-min all-out test, and concluded that changes in intramyocellular pH might not influence *W’* in this particular test setting, and that for short all-out exercise, [PCr] dynamics is more important in determining *W’*. In our constant-load trials at CP, *W’* was supplied to a large extent by anaerobic glycolysis. Therefore, we assume that NaHCO_3_ supplementation increases *W’* in conditions where acidification occurs during exercise. Our result that the estimated *V̇ *O_2_ slow component was not different between the two interventions lends further credence to this notion, although the influence of NaHCO_3_ on the *V̇ *O_2_ slow component remains ambiguous (reduction: [30]; no change: [31]). In our study, the identical *V̇ *O_2_ slow component for both, the NaHCO_3_ and placebo condition, indicated that *V̇ *O_2peak_ was attained at the same point in time. Based on the fact that the depletion of *W’* coincides with the attainment of *V̇ *O_2peak_[32], our results indicate that NaHCO_3_ ingestion did not increase the rate of *W’* utilization but rather *W’* itself. Further support for our assumption comes from another study, where average power in a 60 min cycling time trial was found to be higher with NaHCO_3_ as compared to placebo [33]. During a 60 min time trial, power output will fluctuate around CP with power peaks occurring *e.g.* at the start and during (final) sprints*.* In these occasions, *i.e.* when exercising above CP, *W’* will be reduced. Consequently, a higher *W’* can increase performance during tests of longer duration, especially if pacing strategies are implemented.

We also found that five bolus intakes on five consecutive days did not result in an increase of *T*_lim_ beyond the value observed after the first intake. Thus, multiday administration of NaHCO_3_ did not lead to a cumulative effect on endurance capacity. Accordingly, [HCO_3_^-^], blood pH, and ABE after multiday NaHCO_3_ administration also remained unchanged relative to the initial rise after the first bolus. The most obvious explanation would be that during each CP-trial a certain amount of NaHCO_3_ was used, leading to lower values for [HCO_3_^-^], pH and ABE post *vs.* pre test. During the following 24 h of recovery, the body would then be expected to re-establish the resting values. On the following day, the participants then would start the CP trial at similar (complete recovery) or lower [HCO_3_^-^], blood pH, and ABE (incomplete recovery) relative to the first day, whereby an additional increase in performance would not be expected. Although we did not measure [HCO_3_^-^], pH and ABE before supplementation on the following days, these two described cases can be most likely excluded. The reason for this is that [Na^+^] also did not increase during the consecutive 5 days of NaHCO_3_ supplementation despite the fact that Na^+^, unlike HCO_3_^-^, was not used as a buffer during the CP trials, and that the high amount of ingested Na^+^ could not be used completely through sweating. The predicted sweating rate during exercise of 1 dm^3^∙ h^-1^ water, with a sweat [Na^+^] of 50 mEq∙ dm^3^[34] would have led to a Na^+^ loss of ~0.36 g. This calculated sweat-induced loss of Na^+^ corresponds to ~20% of the daily Na^+^ intake during the placebo intervention. Regarding the substantially higher Na^+^ intake during the NaHCO_3_ intervention, the sweat-induced loss of Na^+^ was negligible during this intervention.

As shown in this study, the NaHCO_3_ intervention led to an increase in [Na^+^] and plasma osmolality after the first bolus administration. This increase was counteracted by an expansion in PV. The increase in PV was to such an extent that pre-exercise blood [HCO_3_^-^], pH, and ABE remained constant during the 5 days of testing. This proposed mechanism of PV expansion has already been described by Máttar *et al.*[35], who showed that plasma [Na^+^] and plasma osmolality were increased after NaHCO_3_ injections in acute cardiac resuscitation. Other mechanisms to counteract increases in [Na^+^] and plasma osmolality comprise a shift of fluid from the intra- to the extramyocellular compartment [36], a stimulation of arginine vasopressin secretion [37], which leads to an intensified water retention from the kidneys [38], and a stimulation of the thirst center whereby more fluid is consumed [37]. In accordance with our results, McNaughton *et al.*[29] found an increase in plasma [Na^+^] after the first of five doses of NaHCO_3_ but no further increase of plasma [Na^+^] on the following days. The elevation of PV in the present study is mirrored by the measured increase in DXA whole-body lean mass. In the DXA two-component soft tissue model, lean mass comprises water, proteins, glycogen and non-bone minerals [27]. As increases in protein, glycogen and non-bone minerals can virtually be excluded (see below), the increase in whole-body lean mass must have resulted from an increase in whole body water, which led to an expansion in PV. Our findings are in accordance with the report of Lands *et al.*[39] who found a significantly higher value for DXA-derived whole-body lean mass after saline infusion given to healthy male participants. Finally, our finding that HR_CLT_ was reduced lends further credence to our result that PV increased as a consequence of NaHCO_3_ supplementation, because PV expansion simultaneously increases stroke volume and reduces sympathetic nervous activity, leaving *V̇ *O_2,CLT_ unaffected [40].

In our study, DXA-derived leg lean mass did neither change between interventions nor over time (Table 2). As with each gram of glycogen stored in muscle tissue 3–4 g of water is bound [28], and body water is present within the lean soft tissue compartment [27], a decrease in leg lean mass in such a short time (2 days) would indicate a loss of glycogen. In turn, glycogen loss would implicate incomplete regeneration, which would manifest itself in a reduced anaerobic work capacity and, accordingly, decreased performance [41]. Since our participants displayed neither a reduction in leg lean mass nor performance, the provided regeneration drink and the participants’ daily nutritional intake were sufficient to restore glycogen from day to day, allowing them to perform maximally on each day.

Our results have at least two practical implications. First, since the [HCO_3_^-^] gradient between intramyocellular compartment and blood did not decrease over time, NaHCO_3_ can be taken daily in multiday competitions or tournaments lasting ≤ 5 d without the risk of reducing performance. Second, the apparent PV expansion in response to the high ion intake (see above) blunted any further increase in [HCO_3_^-^]. If the same mechanism would be true for the chronic supplementation protocol, the effectiveness of this protocol should be questioned, as it seems that [HCO_3_^-^] cannot be increased limitlessly, *i.e.* that it probably reaches a ceiling. The observed ceiling effect was probably based on a metabolic compensation mechanism preventing a disproportionate increase in [HCO_3_^-^]. A respiratory compensation mechanism is unlikely to have occurred in our study because there were no differences between the NaHCO_3_ and placebo intervention for *V̇ *CO_2_ (*P* = 0.903, data not shown) and RER (*P* = 0.556, data not shown) during the resting measurements before the constant-load tests. Of further note is that the standard chronic protocol comprises a daily dose of 0.5 g NaHCO_3_ kg^-1^ body mass [42], which might accentuate the increase in PV and possible side effects. Thus, one adequate dose of NaHCO_3_ administered before the competition should be effective in mediating all of the performance-enhancing effects without the need of a “loading phase”. In this context, our results expand the findings of McNaughton and Thompson [16] as well as Siegler *et al.*[17], who compared different acute and chronic protocols and found that there are no differences between these ingestion protocols with respect to exercise performance.

It may be argued that the present findings could be limited by 1) differences in performance ability throughout the study period and 2) decreasing motivation. Regarding the first point we have shown that CP was neither different between the first and second intervention period nor before the NaHCO_3_ and placebo condition. An increase in CP from the first to the second intervention would have indicated a training effect, whereas a decrease in CP would have indicated incomplete recovery. Hence, we can assume that the participants had the same performance ability throughout the study, allowing a comparison of *T*_lim_ between the two conditions. Regarding the second point, decreasing motivation in a single participant would be evident from a decrease in *T*_lim_ within or between interventions. Considering the single variations in *T*_lim_ irrespective of condition, during which no distinct increases or decreases in *T*_lim_ over time (*i.e.* from the second to the fifth test day) were identified, a decreasing motivation can be excluded for all participants. In addition, *V̇ *O_2,CLT_, *V̇ *CO_2,CLT_ and RER_CLT_ were not different between conditions and days of testing. This indicates that the participants’ effort was constant during the whole study period.

## Conclusion

In conclusion, multiple acute, consecutive day NaHCO_3_ supplementation led to an increase in *T*_lim_ at CP after the first bolus intake. However, while *T*_lim_ remained elevated in the NaHCO_3_ condition, it was not further altered with prolonged NaHCO_3_ supplementation. The increase in *T*_lim_ was accompanied by a higher [HCO_3_^-^] gradient between the blood and the intramyocellular compartment, which stabilized over time in the NaHCO_3_ intervention. In contrast to the theoretical CP-model, where metabolites should reach a steady state during exercise at CP, and consequently, buffer substances should be ineffective in enhancing *T*_lim_, we showed that in practice *T*_lim_ can be increased with NaHCO_3_ supplementation. Furthermore, the high amount of ingested Na^+^ caused a sustained elevation in PV, which inhibited a further increase in [HCO_3_^-^], and consequently limited the performance-enhancing effect. Therefore, this study indicates that NaHCO_3_ can be taken daily in multiday competitions or tournaments to maintain performance ability throughout the whole duration of the competition.

## Abbreviations

ABE: Actual base excess; CP: Critical power; DXA: Dual-energy x-ray absorptiometry; [HCO3-]: Blood bicarbonate concentration; HRCLT: Heart rate during the constant-load trials at CP; NaCl: Sodium chloride; NaHCO3: Sodium bicarbonate; Ppeak: Peak power output; PV: Plasma volume; RERCLT: Respiratory exchange ratio during the constant-load trials at CP; Tlim: Time-to-exhaustion; V̇ O2peak: Peak oxygen uptake; V̇ O2,CLT: *V̇ *O_2_ during the constant-load trials at CP; V̇ CO2,CLT: Carbon dioxide output during the constant-load trials at CP; W’: The finite work capacity above CP

## Competing interests

The authors declare that they have no competing interests.

## Authors’ contributions

SMM, SMG, MT designed the study. SMG and SMM were involved in data collection. SMG, SMM, and MT were involved in statistical analysis and drafted the manuscript. SMM, SMG, SF, UB, CAW, and MT interpreted the data and reviewed the manuscript. All authors read and approved the final manuscript.
